# Outcome of *Candida Parapsilosis* Complex Infections Treated with Caspofungin in Children

**DOI:** 10.4084/MJHID.2016.042

**Published:** 2016-09-01

**Authors:** İlker Devrim, Rana İşgüder, Hasan Ağın, Gökhan Ceylan, Yüce Ayhan, Özlem Sara Sandal, Ferhat Sarı, Ahu Kara, Mine Düzgöl, Gamze Gülfidan, Nuri Bayram

**Affiliations:** 1 Department of Pediatric Intensive Care Unit, Dr. Behcet Uz Children’s Hospital, İzmir, Turkey; 2 Department of Medical Microbiology, Dr. Behçet Uz Children’s Hospital, İzmir, Turkey

## Abstract

**Background:**

We aimed to evaluate the correlation of caspofungin E-tests with the prognosis and response to caspofungin therapy of *Candida parapsilosis* complex bloodstream infections in children hospitalized in a pediatric intensive care unit.

**Methods:**

All children who had *C. parapsilosis* complex bloodstream infections and who were treated with caspofungin were included in this retrospective study. For each patient, the following parameters, including all consecutive blood and central venous catheter (CVC) cultures, the duration between diagnosis and CVC removal, mortality rate, relapses of the *C. parapsilosis* complex infections as well as the demographic features, were recorded.

**Results:**

The central venous catheter survival rate was 33.3% under caspofungin treatment. In 92.4 % of the patients, the negative culture was achieved within a median duration of 12.5 days. The rate of relapses was 18.9%. The overall mortality rate was 37.7% (20 of 53 patients), and the 30-days mortality rate was 7.5% (4 of 53 patients). There was no statistically significant difference between the groups with MIC<2 mg/l and MIC =2 mg/l using CVC survival rate; rate and duration of achieving negative blood culture for *C. parapsilosis complex;* duration of hospital stay; rate and duration of relapses; overall mortality and 30-days mortality.

**Conclusions:**

The beneficial effects of Caspofungin on biofilms has been shown in vivo, while its impact in children for maintenance of CVC was limited in our study but should not be underestimated in children who strongly need the presence of CVCs. The clinicians should weigh their priority for their patients and choose the optimal antifungal therapy for *C. parapsilosis* complex infections in children.

## Introduction

Children in pediatric intensive care units are at risk of developing invasive *Candida* infections (IC).^1^,^2^ *Candida* species are important causative agents of nosocomial bloodstream infections, reported as the fourth most common pathogen in the United States of America and the sixth in Europe.^3^–^5^ The distribution of the *Candida* species has been changing during the last decade. Currently, *C. parapsilosis* is often reported to be the second most common *Candida* species to be isolated from blood cultures, 6 and the incidence of *C. parapsilosis* was reported to be higher in different geographical regions.^3^,^7^–^9^

The guidelines for the treatment of candidemia recommend the removal of central venous catheters (CVC) in the presence of catheter-related bloodstream infections, if possible.^6^,^10^,^11^ Echinocandins are the newest class of antifungal agents, and they interfere with cell wall synthesis by inhibition of the synthesis of 1,3-*β*-D-glucan in the fungal cell wall.^12^–^14^ Caspofungin has been proven to be as effective as, and less toxic than, amphotericin B in the treatment of IC.^15^,^16^ However, caspofungin MICs for *C. parapsilosis* are higher than those for other *Candida* species, with average MIC50 and MIC90 values ranging between 0.85 to 2 mg/ml and 2 to 2.33 mg/ml, respectively.^17^–^22^ Nevertheless, there is insufficient data to support a correlation between echinocandin susceptibility testing^23^ and clinical outcomes, and further, there have been significant interlaboratory variations in MIC results.^24^

The purpose of this study was to evaluate the correlation of caspofungin E-tests with the prognosis and response to caspofungin therapy of *C. parapsilosis* complex bloodstream infections in children hospitalized in the pediatric intensive care unit (PICU).

## Patients and Methods

Dr. Behçet Uz Children’s Hospital is a 400-bed pediatric training hospital with annual outpatient visits exceeding 565,000 in 2011 and approximately 20,000 hospitalizations per year. The PICU of Dr. Behçet Uz Children’s Hospital is a 24-bed unit, with an annual admission rate of about 600 patients. This retrospective study included the patients who were admitted to PICU during the period between December 2011 and December 2013. During this period, 1–3 lumened polyurethane non-tunneled temporary central venous catheters had been used for CVC cases. All admitted patients were identified using both hospital and unit-specific databases.

A favorable response was defined as the documented clearance of *Candida* species from the blood should be a requirement for a successful outcome.^25^ Patients with favorable responses at the end of the intravenous study therapy were considered to have a relapse if invasive candidiasis had recurred after 14 days following negative culture during the follow-up period. Mortality rates were calculated from the initiation of the study through the end of follow-up; mortality was considered to be attributable to *C. parapsilosis* if candidiasis was identified by the investigator as the cause of death, Candida cultures grew within 48 hours of death, or there was histopathologic or cultural evidence of invasive candidiasis at autopsy. The catheter removal was performed under the conditions described by IDSA and ESCMID guidelines.^10^,^11^

The patients with candidemia who were followed-up in PICU and treated with caspofungin [with a dosage of 50 mg/m^2^ daily (after 70 mg/m^2^ on day 1)] were included in the study. Candidemia was defined by isolation of Candida species from the blood samples of the patients hospitalized in the PICU for at least 48 hours. The presence of fever and signs of infection while blood culture samples taken were strictly required for inclusion. For the patients with one or more positive cultures for Candida, the time of the first isolated culture was considered as the date of enrollment in the study. Patients who had been in PICU for less than 48 hours before Candida isolation and with insufficient data in his or her files were excluded from the study group.

### Microbiological Methods

Episodes of candidemia were detected, using standard aerobic and anaerobic blood Culture media in automated blood culture systems (BACTEC 9240 BD Diagnostic Systems, Sparks, MD). The yeast identification was performed using API 20C AUX (bioMérieux, France). In vitro activity of the antifungal agents was determined, using the E-test (bioMérieux, France) with RPMI medium in accordance with the manufacturer’s instructions. The susceptibility interpretive criteria recommended by the Clinical and Laboratory Standards Institute (CLSI), formerly the National Committee for Clinical Laboratory Standards (NCCLS), were used for caspofungin (CLSI M27-A3 reference method).^26^ Isolates were classified as susceptible (S), intermediate (I), or resistant (R) using the following CLSI caspofungin breakpoints: for *C. parapsilosis*, S≤2, I=4, R>=8 mg/liter.^27^

### Patient data

The investigators recorded the following patient data: age, gender, underlying disease and co-morbidities, indications for admissions and presence of CVC. The duration of caspofungin therapy and previous antifungal therapy were recorded. For each patient, the following parameters, including all consecutive blood and CVC cultures, the duration between diagnosis and CVC removal, seven days, 30-days and overall mortality rate (mortality during the first year of follow-up), relapses of the *C. parapsilosis complex* infections as well as the demographic features, were recorded.

### Statistical analysis

Statistical analysis was performed using SPSS, version 15.0 (IBM SPSS, Chicago, Illinois). Quantitative data were shown as the mean ± standard deviation (SD) or the median with interquartile ranges (Q1–Q3). Qualitative variables were expressed as absolute and relative frequencies. Categorical variables were compared using the χ2 test, whereas Student t test or Mann-Whitney U test was applied for continuous variables. A value of p< 0.05 was considered to be significant. Spearman rank correlation test was used for further correlation analysis.

The study was approved by the local ethics committee of Dr. Behçet Uz Children’s Hospital.

## Results

This retrospective study included 53 patients with a median age of 11 months (ranging from 3 months of age to 14 years of age). Thirty patients (56.6%) were male, and 23 patients (43.4%) were female. The associated underlying diseases were as follows: 24 inborn metabolic disease (45.3%); 13 hemato-oncologic malignancies (24.5%); 9 cardiovascular surgery (17%); 6 respiratory diseases (11.3%); and 1 (1.9%) complicated burn. Among 53 patients, 45 (84.9%) patients had CVCs and the median duration of CVC was 39 days (ranging from 7 days to 391 days).

The median duration of *C. parapsilosis* complex isolation after admission to the hospital was 31 days (ranging from 4 days to 825 days). Thirty-eight patients (71.7%) had used empirical fluconazole (6 mg/kg/day) with a median of 7 days (ranging from 2 to 15 days) before *C. parapsilosis* complex isolation.

The MIC values for caspofungin were as follows: 0.5 mg/L in 3 isolates, 1 mg/L in 13 isolates and 2 mg/L in 37 isolates.

### Treatment and outcome

The median duration of caspofungin treatment was 28 days (ranging from 15 to 52 days). The CVC rescue rate with caspofungin treatment was 33.3% (15 of the 45 patients with CVC). In 49 patients (92.4%), the negative culture was achieved within a median duration of 14 days (ranging from 2 to 36 days). The rate of relapses with *C. parapsilosis* complex was 18.9% (10 patients), and the median duration of relapse was 11 days (ranging from 3 to 35 days). Relapses were observed in 8 of 45 (17.8%) patients with CVC and 2 of 8 (25%) patients without CVC at the onset of candidemia (p=0.6). In particular, out of 8 cases of candidemia relapse in patients with indwelling CVC at the onset of infection, it occurred after CVC removal in 6 cases and presence of CVC in the remaining 2 cases. The median duration of candidemia was 14 days and 11.5 days in patients with CVC and without CVC, respectively (no statistically significant difference).

The overall mortality rate was 37.75% (20 out of 53 patients), and the 30-days mortality rate was 7.5% (4 out of 53 patients). In our study, no death was observed during the first seven days after *C. parapsilosis* complex isolation. One patient who died in the 30 days period had negative cultures for *C. parapsilosis* complex, while the remaining three fatal cases were still candidemia. The rate of having negative blood culture was significantly lower in the fatal cases (p=0,001). The CVC was not removed in 15 of 41 patients (36.6%) in the survived group, and all CVC had been removed in the fatal cases. However, no significance was present between these two groups (p>0.05).

### Comparison of clinical outcomes of patients with C. parapsilosis complex candidemia with MIC=2 mg/l and MIC<2 mg/l for caspofungin

There was no statistically significant difference between the groups with MIC<2 mg/l MIC =2mg/l using CVC survival rate, rate, and duration of achieving negative blood culture for *C. parapsilosis complex*, duration of hospital stay, rate and duration of relapses (Table 1). Overall mortality and 30-days mortality was not significantly different between the high MIC group (=2 mg/l) and lower MIC group (<2 mg/l) (Table 1).

A correlation analysis was performed, and no correlation was found between MIC levels and catheter survival rate and duration, for achieving negative blood culture (p=0.659, p=0.27 and p=0.73, respectively).

## Discussion

In this study, we reviewed our experience with caspofungin in the treatment of *C. parapsilosis* complex bloodstream infections in children hospitalized in PICU. Caspofungin was proven to be effective and safe in invasive Candida infections.^28^ Besides other Candida species C. parapsilosis had a different susceptibility profile for echinocandins. Due to the polymorphism at the hotspot region 1 of the FKS1 gene, the MIC values for all echinocandins were higher compared to other species,^29^,^30^ with average MIC50 and MIC90 values ranging between 0.85 to 2 mg/l and 2 to 2.33 mg/l, respectively.^17^–^22^ Moreover, in a previous study from Spain analyzing 360 isolates; 98.5% of the *C. parapsilosis* complex was reported to be susceptible to caspofungin suggesting the possible resistance.^31^ Our findings also supported the higher MIC values and the MIC of *C. parapsilosis* complex in our study accumulated mostly in higher values of 0.75 mg/l.

The patients requiring prolonged use of a central venous catheter or an indwelling device are at an increased risk for infection with *C. parapsilosis.*^6^ In our study, 84.9% of the patients with *C. parapsilosis* complex candidemia had CVC supporting the previous findings.^6^ In one study focusing on the *C. parapsilosis* biofilms caspofungin was reported to have an antimetabolic activity on *C. parapsilosis* depending on their status of maturation.^32^ Despite higher negative culture rates under caspofungin therapy and having caspofungin sensitive *C. parapsilosis* complex isolates, only one-third of the patients’ catheters could be preserved within caspofungin treatment. Within our knowledge, our study was the first report that could give catheter rescue rate in *C. parapsilosis* complex infections in children treated with caspofungin. In a small scale study covering pediatric hematology-oncology patients with *C. parapsilosis* catheter-related bloodstream infections, none of the antifungals, including caspofungin, amphotericin B, and voriconazole, were found to be able to rescue the long-term central venous access device (ports).^33^ Relatively lower rate of preservation of CVC could be due to the biofilm formation. The maturation of biofilms at CVCs might be different in patients whose CVC were rescued or not.^34^ Due to the prolonged stay of CVCs at our center, the possibility of mature biofilm formation might increase throughout the PICU stay-time. Another possible reason for a lower rate could be because the MICs of caspofungin against *C. parapsilosis* biofilms were higher than the MICs against *C. albicans* biofilms.^35^ Caspofungin was reported to reduce the tissue burden of mice infected with isolates with MICs ≤0.5 μg/mL but was less effective against those with MICs of 1 mg/L.^36^ Since the rescue rate was not significantly different in patients with different MICs in our study, biofilm formation seems to be the major obstacle for catheter rescue.

The eradication of the *C. parapsilosis* complex and having a negative culture was observed in 92.4% of the patients and was higher compared to the previous reports.^36^ In one review of caspofungin database, the response rate in *C. parapsilosis* group was reported to be 74% and similar across the other Candida species.^37^ Moreover, the positive culture rate, despite caspofungin treatment, was persistently 17% (12 out of 70 *C. parapsilosis* isolations). However, we had a relatively low rate of rapid clearance of *C. parapsilosis* complex in 72 hours in 5 of the 53 patients, which would bring up additional issues for discussion of caspofungin usage in *C. parapsilosis* complex infections. There was a debate for using echinocandins in the treatment of *C. parapsilosis* infections. In one report from Spain, authors suggested that the initial use of an echinocandin-based regimen does not seem to influence negatively the outcome in *C. parapsilosis* bloodstream infections.^38^ However, there was an editorial comment in the same journal suggesting a questionable indication of the use of echinocandins in some *C. parapsilosis* complex candidemia episodes.^39^ Previous reports showed a 2.5 median days of *C. parapsilosis* fungemia clearance, however, in our study the median duration for achieving negative culture was 12.5 days, suggesting that caspofungin could eradicate the *C. parapsilosis* complex in relatively long-term compared to previous reports.^37^ In the interpretation of the results and the comparison of the various series, the impact of CVC (presence, removal, and timing of removal) should be considered as a crucial factor in the outcome of candidemia.

The mortality from all causes in patients who was treated with caspofungin was reported to be 26% across the combined non*albicans* group and 29% in the *C. albicans* group in previous reports.^37^ However, in this report, the lowest mortality rate was obtained in *C. parapsilosis* among other Candida species and reported as 20%. In our study, the 30-days mortality and overall mortality was 7.5 % and 37.7%, respectively. Our overall mortality rate was higher than the previous reports since the study included critically ill patients hospitalized in PICU; the higher mortality rates were thought to be clinical conditions possibly unrelated to fungal infections. In our view, seven days and 30-days mortality rates would reflect possible deaths attributable to invasive candida infections and were found to be lower compared to the previous reports. Although median duration of the documented clearance of *C. parapsilosis* complex was longer than previous reports, no death was observed during the first seven days, suggesting caspofungin did control the *C. parapsilosis* infection before eradicating the agent totally. One of the most important findings in our study, 30 days and the over-all mortality rate was not different under MIC levels under or equal to 2 mg/l. Only one patient died despite having negative blood cultures, and 30-days mortality was higher in patients who had persistent candidemia under antifungal therapy, suggesting that microbiological eradication should be achieved for a favorable response. Regarding our outcome results; the increased MIC values for echinocandins in *C. parapsilosis* should be carefully evaluated. However, the clinical response could be different from the susceptibility results.

The present study has some limitations. First of all, the study was retrospectively designed, and no randomization and/or comparison were performed between echinocandins and azoles. Secondly, the susceptibility of *C. parapsilosis* complex had been determined with caspofungin e-test, and breakpoints were used according to the CLSI M27-A3 reference method for caspofungin, 24 which was recommended during the study period. Currently, EUCAST has suggested that laboratories report anidulafungin and micafungin MIC’s.^40^–^42^ However, no data was available for anidulafungin or micafungin MICs in our study. Pfaller, et al. suggested that caspofungin susceptibility test should be improved to overcome its pitfalls^43^ and since caspofungin is the only echinocandin class antifungals at present licensed for usage in children in our country, caspofungin e-tests had been currently used in our clinic. In addition, there were limited data on caspofungin usage in children for *C. parapsilosis* complex infections and our study has provided additional data including catheter rescue rates, microbiologic and clinical response plus mortality rates.

## Conclusion

The treatment of *C. parapsilosis* complex candidemia remains a challenging problem for clinicians due to crude mortality, the formation of biofilms in children with central venous catheters and higher MICs for echinocandins. The beneficial effects of caspofungin on biofilms has been shown in vivo, while its impact in children for maintenance of CVC was limited in our study but should not be underestimated in children who strongly need the presence of CVCs. The clinicians should weigh their priority for their patients and choose the optimal antifungal therapy for *C. parapsilosis* complex infections in children.

## Figures and Tables

**Table 1:** Comparisons of clinical outcomes of patients with *C. parapsilosis* complex candidemia with MIC=2 mg/l and MIC< 2 mg/l for caspofungin.

Variables	MIC <2	MIC =2	P

Catheter, n (%)			
Recovered	4 (30.8)	11 (34.4)	1.0
Removed	9 (69.2)	21 (65.6)	

Presence of achieving negative blood culture, n (%)			
Yes	16 (100)	33 (89.2)	0.3
No	0	4 (10.8)	

Negative blood culture time (days), median (min–max)	15 (2–28)	13 (3–36)	0.79

Relapse, n (%)			
Yes	4 (25)	6 (16.2)	0.46
No	12 (75)	31 (83.8)	

Relapse time (days), median (min–max)	14 (3–33)	11 (3–35)	0.96

Duration of hospital stay (days), median (min–max)	65 (26–277)	67 (15–484)	0.61

Overall mortality, n (%)	6 (37.5)	14 (37.8)	0.98

30-days mortality, n (%)	0	4 (10.8)	0.3
